# Chronic post-surgical pain after knee arthroplasty: a role of peripheral nerve blocks

**DOI:** 10.3389/fmed.2023.1335405

**Published:** 2024-01-11

**Authors:** Svetlana Sreckovic, Nebojsa Ladjevic, Biljana Milicic, Goran Tulic, Darko Milovanovic, Marija Djukanovic, Marko Kadija

**Affiliations:** ^1^Centre of Anaesthesia and Resuscitation, University Clinical Centre of Serbia, Belgrade, Serbia; ^2^Clinic for Orthopaedics Surgery and Traumatology, University Clinical Centre of Serbia, Belgrade, Serbia; ^3^Medical School, University of Belgrade, Belgrade, Serbia; ^4^Department of Medical Statistics and Informatics, Belgrade, Serbia; ^5^School of Dental Medicine, University of Belgrade, Belgrade, Serbia

**Keywords:** peripheral nerve block, chronic pain, adductor canal block, IPACK block, knee arthroplasty

## Abstract

**Introduction:**

Peripheral nerve blocks are an efficient method of pain control after total knee arthroplasty (TKA), but there is no report of their impact on chronic post-surgical pain (CPSP).

**Methods:**

This prospective observational study aimed to assess adductor canal block (ACB) and IPACK block (blocks vs. no blocks) on opioid consumption, postoperative pain score, chronic post-surgical pain 2 years after TKA.

**Results:**

166 patients (82 vs. 84) were analyzed. Opioid consumption was less in the group with blocks (9.74 ± 3.87 mg vs. 30.63 ± 11.52 mg) (*p* < 0.001). CPSP was present in 20.24% of patients in the group without blocks and 6.1% of patients with blocks (*p* = 0.011). Predictor variables of CPSP included pain before surgery (cut-off of 5.5), pain at rest (cut-off of 2.35), pain during active movement (cut-off: 2.5), and opioid consumption (cut-off: 8 mg).

**Conclusion:**

Peripheral nerve blocks provide adequate analgesia, significantly decrease opioid consumption, improve functional outcomes, and reduce CPSP 2 years after surgery.

## 1 Introduction

Total knee arthroplasty (TKA) is a standard surgical procedure in the final stages of osteoarthritis associated with intense postoperative pain, especially in the first 24 h postoperatively and during active joint movements (1, 2). After this procedure, different pain management strategies are suggested to provide adequate analgesia without muscle weakness, to enable early rehabilitation, and to prevent chronic pain (3–6). Adductor canal block (ACB) and infiltration in the space between the popliteal artery and the capsule of the posterior knee (IPACK block) are described as an effective method of pain management in these patients, which significantly leads to a reduction in opioid consumption, pain, and improve knee functioning in the immediate postoperative period (5, 6). However, there is no report of their impact on chronic post-surgical pain (CPSP). CPSP after TKA was experienced in up to 44% of patients, and 15% were in severe pain, affecting the quality of life, causing dissatisfaction, and becoming one of the reasons for revision surgery (7–9). One of the predictors of CPSP is postoperative pain intensity, mainly provoked by movement (10, 11). Some studies suggest that pain duration is equally significant (3, 4). Various pathophysiological mechanisms, including sensitization at the site of surgery and the spinal and supraspinal levels, are responsible for developing chronic pain (12).

The estimation of outcomes after TKA is very challenging. Implant survival was the most commonly referenced measure of success after knee arthroplasty. Patient-reported outcome measures (PROMs) have been widely used to assess pain and function after TKA (13–16).

This single-center, observational study aimed to assess the impact of ACB and IPACK block on the incidence of CPSP and functional recovery 2 years after TKA.

## 2 Methods

### 2.1 Patients and study design

The study was carried out by the principles of the Helsinki Declaration. After approval by the Ethics Committee of the University Clinical Centre of Serbia (No 361/14; No 340/1), and obtaining written informed consent 300 patients were included in the study. All patients underwent elective TKA from January 2018 to April 2020, in the Clinic for orthopedics surgery and traumatology, University Clinical Centre of Serbia.

This study was written following the Strengthening the Reporting of Observational Studies in Epidemiology (STROBE) guidelines.

Inclusion criteria were: primary unilateral TKA, age of 40–90 years, ASA I-III, type of implant (NexGen® Complete Knee Solution, Zimmer Biomet, Indiana, United States), no history of ongoing opioid treatment within 30 days before surgery. Exclusion criteria were: incomplete data, psychological, emotional or neurological conditions that may jeopardize postoperative rehabilitation (drug or alcohol abuse, mental illness, neurological diseases-Parkinson’s disease, multiple sclerosis, etc.).

### 2.2 Intervention

All patients underwent a unilateral cemented TKA without patella resurfacing using a tourniquet, inflated to 100–150 mmHg over systolic blood pressure. Spinal anesthesia was performed using levobupivacaine 0.5% up to 3 mL at the level of the L3-L4. If a patient refused spinal anesthesia or there was its contraindication, general anesthesia was induced with midazolam 0.05 mgkg-1, fentanyl 3mcgkg-1, propofol 1.5–2.0 mgkg^−1^, and cisatracurium 0.2 mgkg^−1^. Anesthesia was maintained with sevoflurane at a minimum alveolar concentration of 1. Postoperatively, multimodal analgesia regime depended on the anesthesiologist’s affinity and included: (a) non-opioid analgesics (paracetamol 1 g iv. every 8 hours and ketorolac 30 mg iv. every 8 hours); (b) opioid analgesics (tramadol 100 mg iv., morphine 4–6 mg iv/im/sc, and tapentadol 50–100 mg *per os*) and (c) combination of ACB and IPACK block. This combination of nerve blocks was performed by one anesthesiologist. ACB was performed using a linear probe of 10–12 MHz, 15 mL of 0.33% levobupivacaine (10 mL of 0.5% levobupivacaine and 5 mL of 0.9% sodium chloride) injected lateral to the femoral artery at the midpoint of the adductor canal beneath the sartorius muscle. The IPACK block was performed with a curved (2–5 MHz) transducer positioned over the medial thigh. After visualizing the femoral shaft, popliteal artery, and posterior space of the femoral shaft, 15 mL of 0.33% levobupivacaine was injected.

Patients were divided into two groups based on the administration of peripheral nerve blocks, the first group with blocks(АCB and IPACK block) and the second without blocks.

### 2.3 Postoperative outcomes

#### 2.3.1 Pain intensity

Pain intensity was measured using the Numerical Rating Scale (NRS), at 1 hour, two, three, four, six, eight, 12 h, 16 h, 20 h, and 24 h postoperatively. NRS is commonly used to assess pain severity at that moment in time using a 0–10 scale, with zero meaning *no pain* and 10 meaning *the worst pain imaginable.* Patients were given an intravenous bolus of morphine as *rescue* analgesia when pain at rest was more than three or more than five when they were moving, breathing deeply, or coughing. Morphine in a dose of 1 mg was given every 10 min until the pain intensity was reduced. NRS estimation was also performed during early rehabilitation, five hours after surgery. It included active movements of the operated leg and full flexion of the knee and foot: I-flexion of the foot; II-partial flexion of the knee; III-full flexion of the knee and foot; IV-raising the leg in full extension and holding it for 10 s. Opioid requirements were measured by converting the 24-h opioid consumption into a standardized morphine milligram equivalent (MME).

#### 2.3.2 Functional outcomes

The patient’s opinion about the operative knee and associated problems were assessed by *Knee Injury and Osteoarthritis Outcome Score* (KOOS) 2 years after surgery. The questionnaire includes five subscales: *Symptoms and stiffness* (seven items); *Pain* (nine items); *Function, daily living* (17 items); *Function, sports, and recreational activities* (five items) and *Quality of Life* (four items). All items have calculated as the sum and transformed to a 0–100 scale, with zero representing severe knee problems and 100 representing no knee problems. Scores between 0 and 100 represent the percentage of the total possible score achieved. Assessment of patients’ ability to forget about the artificially implanted knee joint in performing daily activities was evaluated by the *Forgotten Joint Score* (FJS) 2 years after surgery. Every question is scored from 1 (never) to 5 (mostly) according to the selected response, and the raw score ranges from 12 to 60. The raw score is linearly transformed to a 0–100 scale and then reversed to obtain the final score (Final score = 100 - ((sum(item01 to item12) - 12)/48*100)). Higher scores indicate that the patient is less aware of artificial joint when performing daily activities.

#### 2.3.3 Postoperative complications

Low molecular weight heparin (LMWH) was administrated 12 h before surgery in all patients. The dose was prophylactic or therapeutic, adjusted to the patient’s need, according to the value of anti-Xa, and continued in the next 30 days after surgery. Discharge criteria were good health condition with no wound exudation and the flexion angle of the operative knee >90°. Nausea, vomiting, itching, sleepiness, delayed wound healing, drainage or swelling, deep venous thrombosis, and other cardiovascular, neurovascular, or cerebrovascular complications during hospitalization were recorded. The persistence of CPSP in the operative knee was assessed by pain specialists, according to the International Classification of Diseases, Eleventh Revision (ICD-11) and intensity was estimated using NRS 2 years after surgery (17).

### 2.4 Statistical analysis

For normal distribution data testing, the Kolmogorov–Smirnov and Shapiro–Wilk tests were used. Descriptive methods (percent, mean, median, standard deviation (SD)) were used to summarize the data. The Pearson *Χ*^2^ test; Fisher exact test; Kruskal Wallis test; Wilcoxon rank sum test was used to test differences between groups depending on the nature of the examined parameters. The receiver operating characteristic (ROC) curve methods were applied (AUC ROC-area under the ROC curve according to DeLongs method; likelihood ratio test for AUC ROC; the best cut-off value for these parameters was set as value with maximum sensitivity and specificity) to examine the discriminative potential of factors relevant to the presence/absence of CPSP 2 years after TKA. All tests were two-sided, and statistical significance was considered at *p* < 0.05. The statistical analysis was done with the program R version 3.3.2 (2016-10-31) - “Sincere Pumpkin Patch”; Copyright (C) 2016 The R Foundation for Statistical Computing; Platform: x86_64-w64-mingw32/x64 (64-bit) (available: www.r-project.org; downloaded: January 21, 2022).

## 3 Results

**Figure 1:**
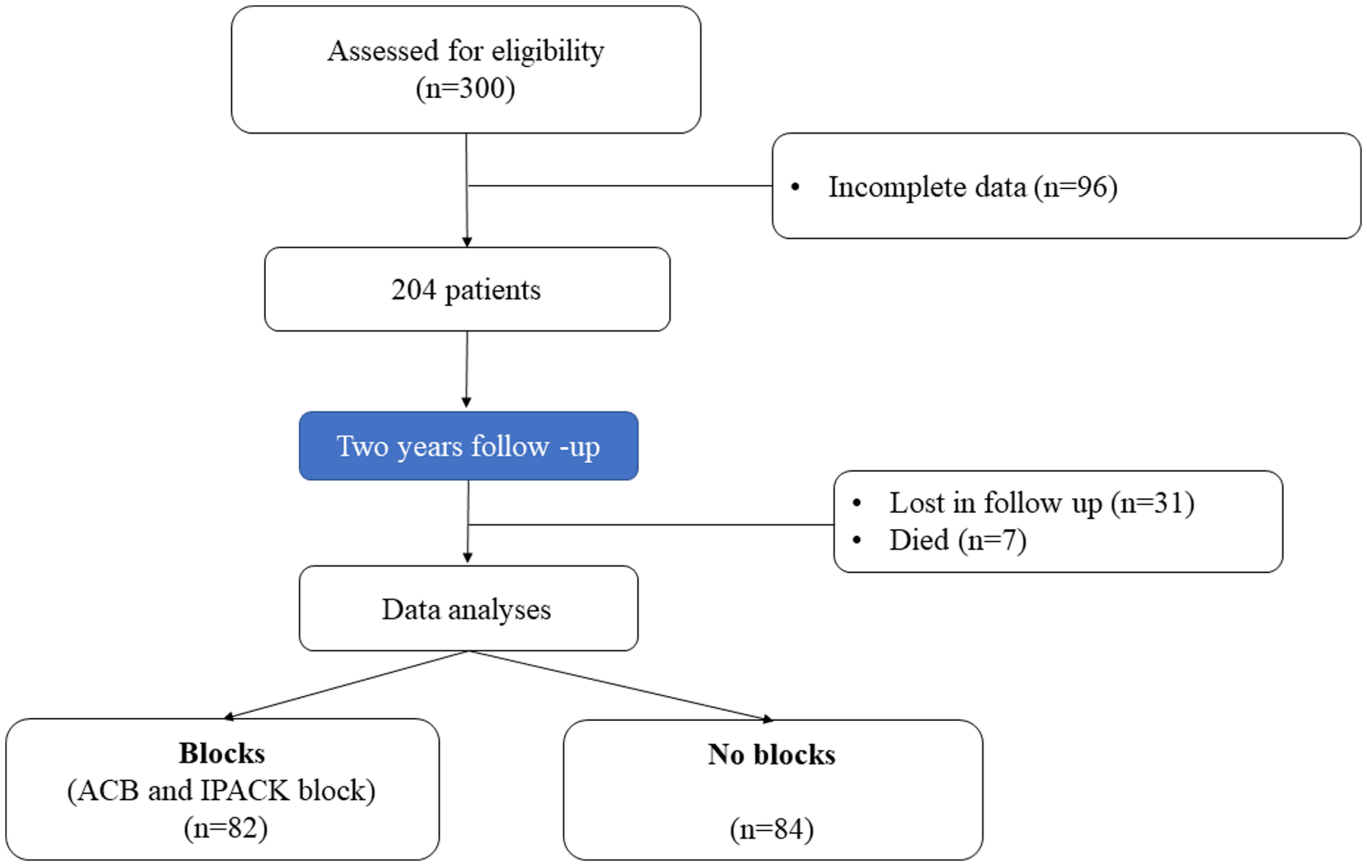
Patient selection and study flow.

This prospective observational study included 166 patients with complete medical data, followed up 2 years after elective TKA. Ninety-six patients had incomplete data, 31 were lost, and seven died within 24 months of follow-up. Data for 82 patients with blocks (ACB and IPACK block) in the first group and 84 in the second without blocks were analyzed (Figure 1). There was no difference between groups regarding patient characteristics- age, sex, ASA status, BMI, and the type of anesthesia. All patients were in pain for more than 3 months before surgery, but there was no difference between groups in pain intensity and the number of painful joints affected (Table 1). Also, there was no difference between groups in the number of patients in pain and its intensity 1 hour after surgery (Table 2). The group with blocks experienced less pain at rest (1.19 ± 0.73 vs. 3.10 ± 1.08 p<0.001) and at the other time points in the first 24 h (Table 2). Also, there was a statistical difference between groups in pain during coughing and active movements of the operated leg (Table 2). In the group with blocks, 68.29% of patients were in pain during flexion of the foot with an intensity of 1.55, while in the group without blocks, 95.25% had a pain intensity of 3.75. Differences between groups also existed during partial flexion of the knee and full extension of the knee and foot. All patients with blocks could raise the leg in full extension and hold it for 10 s, and only eight patients in the group without blocks could perform the same. Pain intensity during this activity was higher in the group without blocks (2.54 ± 1.16vs 8.62 ± 3.11) (Table 2). In the first 24 h after surgery, in the group with blocks, 23.2% of patients needed opioids.

**Table 1:** Patient characteristics.

Characteristics	Blocks	No blocks	*p* value
*Age (y)*			
Mean (SD)	68.51 (8.7)	67.14 (7.62)	0.421
Median (range)	68(50–90)	67 (43–90)	
*Sex - n (%)*			
Male	30 (36.59%)	24 (28.57%)	0.349
Female	52 (63.41%)	60 (71.43%)	
*Weight (kg)*			
Mean (SD)	84.11 (15.06)	81.19 (13.91)	0.098
Median (range)	85(60–120)	80(56–115)	
*Height (m)*			
Mean (SD)	1.72 (0.09)	1.71(0.08)	0.346
Median (range)	1.72(1.5–1.91)	1.69(1.52–1.92)	
*BMI (kg/m^2^)*			
Mean (SD)	28.32 (3.39)	27.59(3.88)	0.502
Median (range)	28.38(22.23–37.78)	27.47(20.08–38.77)	
*ASA physical status - n (%)*			
ASA I	1 (1.22%)	1 (1.19%)	0.51
ASA II	38 (46.34%)	46 (54.76%)	
ASA III	43 (52.44%)	37 (44.05%)	
*Type of anesthesia - n (%)*			
General	31 (37.80%)	26 (30.95%)	0.444
Spinal	51 (62.20%)	58 (69.05%)	
*In pain before surgery (during ≥ 3 months)*			–
In the knee for surgery	80 (97.6%)	83 (98.8%)	0.983
In the knee & another place	2 (2.4%)	1 (1.2%)	
*Pain before surgery* (NRS) – mean (SD)	6.11(1.15)	6.33(1.15)	0.21
Total	82 (100%)	84 (100%)	–

BMI-body mass index; ASA- American Society of Anesthesiologists; NRS- Numerical Rating Scale.

**Table 2:** Postoperative pain score.

Characteristics	In pain – *n* (%)			Pain (NRS) – mean (SD)		
	Blocks	No blocks	*p* value	Blocks	No blocks	*p* value
*Pain after surgery, at rest*						
1 h	41 (50)	35 (41.67)	0.28	2.34 (1.77)	2.09 (0.74)	0.46
2 h	44 (53.66)	80 (95.24)	<0.001	2.07 (1.23)	2.84 (1.5)	0.004*
3 h	52 (63.41)	84 (100)	<0.001	2 (1.41)	3.54 (1.96)	<0.001
4 h	59 (71.95)	82 (97.62)	<0.001	1.92 (1.25)	3.77 (1.95)	<0.001
6 h	53 (64.63)	80 (95.24)	<0.001	1.53 (0.67)	3.71 (1.92)	<0.001
8 h	54 (65.85)	82 (97.62)	<0.001	1.78 (1.14)	4.02 (1.94)	<0.001
12 h	52 (63.41)	81 (96.43)	<0.001	1.9 (1.5)	4 (1.91)	<0.001
16 h	45 (54.88)	82 (97.62)	<0.001	1.51 (0.79)	3.34 (1.69)	<0.001
20 h	47 (57.32)	81 (96.43)	<0.001	1.51 (0.59)	3.15 (1.57)	<0.001
24 h	47 (57.32)	65 (77.38)	0.006*	1.53 (0.65)	3.4 (1.64)	<0.001
Within 24 h	75 (91.46)	84 (100)	0.006*	1.19 (0.73)	3.10 (1.08)	<0.001
*Pain during activity*						
Flexion of the foot	56 (68.29)	80 (95.24)	<0.001	1.55 (0.69)	3.75 (1.89)	<0.001
Partial flexion of the knee	58 (70.73)	82 (97.62)	<0.001	1.57 (0.7)	4.2 (1.82)	<0.001
Full flexion of the knee and foot	64 (78.05)	84 (100)	<0.001	1.78 (0.88)	8.77 (2.03)	<0.001
Raising the leg in full extension and holding it for 10 s, done	82 (100)	8 (9.52)	<0.001	2.54 (1.16)	8.62 (3.11)	<0.001
*Pain during coughing*						
In pain	5 (6.1)	55 (65.48)	<0.001	1.6 (0.55)	3.75 (1.54)	0.001*
Total	82 (100)	84 (100)	-	82 (100)	84 (100)	-

NRS- Numerical Rating Scale; ^*^p < 0.05.

During this period, opioid consumption was less in this group (9.74 ± 3.87 vs. 30.63 ± 11.52) (Table 3). In the following 24 h, 69 (84.1%) patients in the group with blocks were free of opioids, and without blocks 43 (51.2%) (*p* < 0.001), but without difference in the dose of opioids (Table 3).

**Table 3:** Postoperative opioid consumption.

Characteristics	Patients who needed opioids – N (%)			Dose of opioids (mg) – mean (SD)		
	Blocks	No blocks	p value	Blocks	No blocks	*p* value
*Opioids consumption*						
Within 24 h	19 (23.17%)	84 (100%)	<0.001	9.74 (3.87)	30.63 (11.52)	<0.001
24-48 h	13 (15.85%)	41 (48.81%)	<0.001	11.62 (5.38)	13.9 (4.94)	0.189
48 h-72 h	2 (2.44%)	1 (1.19%)	0.983	10 (0)	20 (0)	0.496
Total	82 (100%)	84 (100%)	–	82 (100%)	84 (100%)	–

Two years after surgery, the KOOS score was statistically different between groups. The higher score was in the group with blocks (92.61 ± 10.85 vs. 81.81 ± 12.28 *p* < 0.001) and in all its subscales (Table 4). FJS 2 years after surgery was significantly higher in the group with blocks (92.52 ± 12.43 vs. 78.72 ± 13.94, *p* < 0.001) (Table 4). In the group with blocks, 91.5% of patients had FJS ≥ 22, while without blocks 59.5% (*p* < 0.001) (Table 4).

**Table 4:** KOOS and FJS 2 years after knee arthroplasty.

Characteristics, 2 years after surgery	Blocks	No blocks	*p* value
*KOOS (%)*			
Mean (SD)	92.61 (10.85)	81.81 (12.28)	<0.001
Median (Range)	94.5 (38–100)	83.5 (38–100)	
*Symptoms + Stiffness (%)*			
Mean (SD)	92.83 (12.14)	84.13 (14.63)	<0.001
Median (Range)	96 (25–100)	89 (8–100)	
*Pain (%)*			
Mean (SD)	93.77 (10.19)	74.19 (14.87)	<0.001
Median (Range)	97 (28–100)	78 (25–100)	
*Function, daily living (%)*			
Mean (SD)	92 (11.46)	81.88 (14.05)	<0.001
Median (Range)	94 (35–100)	85 (25–100)	
*Function, sports, and recreational activities (%)*			
Mean (SD)	91.38 (11.87)	79.1 (13.27)	<0.001
Median (Range)	95 (35–100)	80 (30–100)	
*Quality of life (%)*			
Mean (SD)	96.26 (10.23)	88.9 (11.65)	<0.001
Median (Range)	100 (31–100)	91 (44–100)	
*FJS total (%)*			
Mean (SD)	92.52 (12.43)	78.72 (13.94)	<0.001
Median (Range)	95.83 (33.33–100)	81.25(29.17–97.92)	
*FJS categories – N (%)*			
FJS ≥ 22	75 (91.5%)	50 (59.5%)	<0.001
FJS < 22	7 (8.5%)	34 (40.5%)	
Total	82 (100%)	84 (100%)	–

KOOS- Knee Injury and Osteoarthritis Outcome Score; FJS- Forgotten Joint Score.

**Figure 2:**
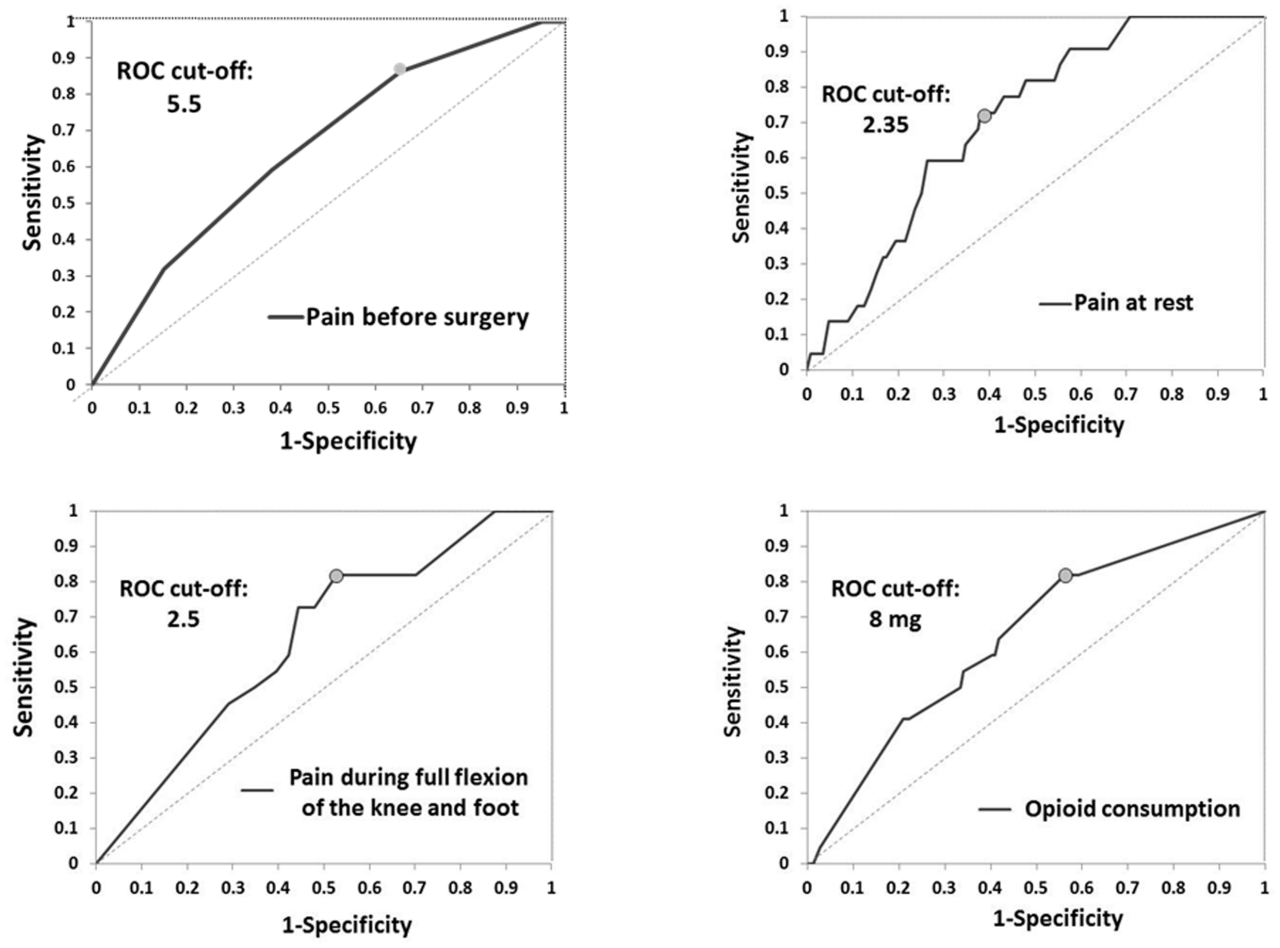
ROC curves.

In the group without blocks, postoperative complications, nausea (*p* < 0.001), and sleepiness (*p* < 0.001) were more often (Table 5). Vomiting, itching, wound infection, DVT (above and below knee), and pulmonary embolism were not present in either group. One patient had a foot drop in the first group as a complication of performed blocks (Table 5) (18). During 24 months after TKA, there was no readmission to the hospital, and seven patients died in both groups (Table 5). Two years after TKA, CPSP was present in 20.24% of patients in the group without blocks and 6.1% of patients with blocks (*p* = 0.011) without differences in pain intensity (Table 5). Potential predictor variables of CPSP were pain before surgery (cut-off of 5.5), and for the first 24 h after surgery pain at rest (cut-off of 2.35), pain during active movement (cut-off: 2.5), and opioid consumption (cut-off: 8 mg) (Table 6; Figure 2).

**Table 5:** Postoperative complications.

Characteristics	Blocks	No blocks	*p*-value
*Postoperative complications*			
*Chronic post-surgical pain*			
Presence-*n* (%)	5 (6.1%)	17 (20.24%)	0.007^*^
Pain- NRS			
Mean (SD)	3.8 (0.45)	3.65 (0.61)	0.18
Median (Range)	4 (3–4)	4 (3–5)	
*Nausea, n (%)*	0 (0%)	24 (28.6%)	<0.001
*Sleepiness, n (%)*	4 (4.88%)	49 (58.33%)	<0.001
*Foot drop, n (%)*	1 (1.22%)	0 (0%)	0.309
*Wound drainage, n (%)*	1 (1.22%)	1 (1.19%)	0.986
*Urinary tract infection, n (%)*	2 (2.44%)	3 (3.57%)	0.669
*Characteristics, 2 years after surgery*			
*24-month mortality, n (%)*	3 (3.66%)	4 (4.76%)	0.724
Total, n(%)	82 (100%)	84 (100%)	–

NRS- Numerical Rating Scale; ^*^- *p* < 0.05.

**Table 6:** Results of the ROC analysis.

Characteristics	Area Under the ROC curve		ROC cut-off value^c^		
	AUC ROC^a^ (95%CI)	Likelihood ratio test^b^	Cut-off value	Sensitivity (95% CI)	Specifity (95% CI)
*Pain before surgery (NRS)*	65.23 (53.72–76.74)	0.0155*	5.5	34.72 (27.08–43.06)	86.36 (72.73–100)
*During the first 24 h after surgery*					
Pain at rest (NRS)	70.57 (60.74–80.39)	0.002*	2.35	72.73 (54.55–90.91)	61.81 (54.17–70.14)
Pain during flexion of the foot (NRS)	63.21 (51.3–75.12)	0.105	–	–	–
Pain during partial flexion of the knee (NRS)	63.59 (52.16–75.02)	0.131	–	–	–
Pain during full flexion of the knee and foot (NRS)	63.54 (52.24–74.84)	0.045*	2.5	81.82 (63.64–95.45)	47.22 (39.58–55.56)
Opioid consumption during the first 24 h (mg)	64.63 (52.82–76.44)	0.032*	8	81.82 (63.64–95.45)	43.75 (35.42–52.08)

a Area Under the ROC curve (DeLong’s method).

b Likelihood ratio test for AUC ROC.

c Value with maximum sensitivity and specificity; NRS- Numerical Rating Scale; ^*^p<0.05.

## 4 Discussion

In this study, we assessed opioid consumption, functional outcomes, and CPSP in the case of peripheral nerve blocks and predictor variables of CPSP. Our results suggest that ACB and IPACK block provide adequate analgesia at different time points, significantly reducing opioid consumption and reducing postoperative complications. 23.2% of patients with blocks had needed opioids in the first 24 h with a dose of less than 10 mg. Also, this study showed that this combination of blocks improves functional outcomes by providing higher KOOS and FJS 2 years after surgery. Patients in the group without blocks were more likely to develop CPSP 24 months after TKA. Potential predictor variables of CPSP were pain before surgery (cut-off of 5.5), and for the first 24 h after surgery: pain at rest (cut-off of 2.35), pain during active movement (cut-off: 2.5), and opioid consumption (cut-off: 8 mg).

Peripheral nerve blocks are part of the multimodal analgesia regimen following TKA. ACB is a part of this regimen, preserving quadriceps strength, and enhancing recovery (4, 19, 20). The analgesic efficiency of this block is mainly the result of blocking the saphenous nerve and the nerve to the vastus medialis, which also play a substantial role in knee joint innervation (21). Administering a high volume of a local anesthetic near the end of the adductor canal could unintentionally block other nerves close to the proximal or distal end of the canal (22). But continuous ACB does not have the benefit of single-shot ACB (23). Zhang et al., showed that ACBs do not provide better pain control than LIA (75 mL total volume included 150mg of ropivacaine, 30mg of ketorolac, adrenaline 200μg and 10mg of morphine), while their combination had been recommended after TKA (24). Pain occurring in the posterior and lateral aspects of the knee after TKA is not covered by the ACB. A new ultrasound-guided technique has been devised that comprises infiltration of the local anesthetic between the popliteal artery and capsule of the posterior knee and provides effective analgesia to the posterior aspect of the knee without affecting muscle strength (25). Combining ACB with IPACK block provides adequate analgesia to the anterior and posterior aspects of the knee without affecting muscle strength (5, 6, 26). In the meta-analysis, Hussain et al. reported that in the absence of LIA, adding IPACK to ACB reduces pain for up to 24 h, enhances functional recovery, and does not support the addition of IPACK to ACB when LIA is routinely administered (27). However, for opioid-sparing effects and for pain scores different minimally clinically important difference (MCIDs) has been used in most studies (28). Laigaard et al. concluded that median clinician-perceived MCIDs in postoperative pain management were 10 mg iv morphine equivalents or 40% of opioid consumption and 15-18 mm or 30% for pain scores (28). Different analgesia regimens enable most patients to be at low risk for moderate or severe pain (29). The use of rescue analgesics for a minor reduction in pain scores and opioid consumption has been dampening, and the patients with higher baseline pain scores may be more responsible for treatment effects (29). Our study showed that if ACB and IPACK block were applied, some patients were not in pain, and for those who needed opioids, the dose was less than 10 mg iv in the first 24 h postoperatively. Between our groups during the first hour postoperatively, there were not any differences, which can be explained by the duration of spinal anesthesia or good analgesia during general anesthesia. Nausea and sleepiness, as opioid-induced side effects, were in the group without blocks as effects of higher opioid postoperative consumption.

Estimation of the outcome after TKA changes through time. Improvements in surgical techniques and implant design made patient satisfaction evolving the most referenced measure of success (30, 31). However, approximately 20% of patients after TKA are dissatisfied with the outcome, and up to 34%, experienced moderate to severe pain 3 months after surgery (32, 33). CPSP is the most common cause of dissatisfaction and one of the reasons for revision surgery after TKA (32). Sakellariou et al. showed that 39 % of patients had persistent pain ranging from 3–5 out of 10 a year after TKA (33). While Lui et al. included 1,030 patients after TKA and showed that the rate of persistent pain after TKA was 53% 1 year after surgery (34). Our study showed that 2 years after TKA, CPSP was present in 6% of patients with ACB and IPACK blocks and 20% of patients without blocks without difference in pain intensity. Various factors have been identified as predictive factors of CPSP. Several single gene mutations have been identified as potential risk factors for increased sensitivity but without powerful predictive value for CPSP. Biological characteristics usually include age and sex, where younger adults and female sex patients are more prone to developing CPSP. Also, patients with increased BMI and persistent comorbidities are at greater risk (28–35). Psychological factors that could have a greater impact on the development of CPSP include depression, sleep disorders, fear of undergoing surgery, anxiety, and a tendency to exaggerate surgery outcomes. Socioeconomic factors usually include lower education levels, marital status, where unmarried patients are at greater risk, smoking, and unemployment (35–42).

Preoperative pain scores and more severe acute postoperative pain were associated with a higher risk of CPSP after TKA (10, 11, 43). Our study showed that risk factors for CPSP are: pain before surgery (higher 5.5 NRS), pain at rest (higher than 2.35 NRS), and during active movement (higher than 2.5 NRS), and opioid consumption of 8 mg. Although there is little evidence of the type and impact of perioperative interventions that reduce CPSP, our results imply the importance of perioperative pain control on the occurrence of CPSP.

KOOS score in the group without blocks was mainly affected by pain. FJS, a newer and more sensitive score, registers the differences in the functioning of more active patients after TKA. Recently, Clement et al. reported that a postoperative FJS of 22 or more, was predictive of achieving a patient-acceptable symptom state (44). Our study showed that the group with ACB and IPACK blocks had 91.5% of patients with FJS ≥ 22, while in the group without blocks, there were only 59.5% (*p* < 0.001).

The limitations of our study include the fact that this was a single-center design. Also, we did not have preoperative values for KOOS and FJS and estimation of psychological factors such as expected pain, depression, sleep disorders, fear of undergoing surgery, anxiety, and a tendency to exaggerate surgery outcomes that could influence recovery and CPSP. The patients in the study group did not have a uniform rehabilitation program, which could also affect our results. Our study provides a basis for further validation in randomized control studies.

## 5 Conclusion

The combination of ACB and IPACK block provides adequate analgesia at various time points postoperatively, enabling early rehabilitation, significantly decreasing opioid consumption, improving functional outcomes by providing higher KOOS and FJS and reducing CPSP 2 years after surgery. Pain intensity before surgery at less than 5.5, at rest at less than 2.35, pain during active movement at 2.5, and opioid consumption at less than 8 mg reduce the incidence of CPSP. Randomized controlled trials with long-term follow-up are needed to confirm the beneficial effects of ACB and IPACK block after TKA.

## Data availability statement

The raw data supporting the conclusions of this article will be made available by the authors, without undue reservation.

## Ethics statement

The studies involving humans were approved by Ethics Committee of University Clinical Centre of Serbia. The studies were conducted in accordance with the local legislation and institutional requirements. The participants provided their written informed consent to participate in this study.

## Author contributions

SS: Conceptualization, Data curation, Formal analysis, Investigation, Methodology, Writing – original draft, Writing – review & editing. NL: Conceptualization, Supervision, Validation, Writing – review & editing. BM: Data curation, Methodology, Software, Writing – review & editing. GT: Conceptualization, Supervision, Writing – review & editing. DM: Investigation, Methodology, Validation, Writing – original draft. MD: Data curation, Investigation, Writing – original draft. MK: Conceptualization, Supervision, Validation, Writing – review & editing.
